# Perceived benefits and barriers to health behaviors after transarterial chemoembolization in patients with hepatocellular carcinoma: a qualitative study based on the health belief model

**DOI:** 10.3389/fmed.2026.1821409

**Published:** 2026-06-22

**Authors:** Yujun Jiang, Wenxi Guo, Yangyang Liu, Wang Feng, Kexin Jiang, Xiaolin Wang

**Affiliations:** 1 School of Nursing, Chongqing Medical University, Chongqing, China; 2 Department of Radiology, The Second Affiliated Hospital of Chongqing Medical University, Chongqing, China

**Keywords:** health behaviors, health belief model (HBM), liver cancer, nursing, qualitative study, transarterial chemoembolization

## Abstract

**Objective:**

Grounded in the Health Belief Model (HBM) as a heuristic framework, this study aimed to characterize the typologies and distinctive features of benefit-barrier perceptions regarding health behavior adherence among liver cancer patients following transarterial chemoembolization (TACE), focusing specifically on the core constructs of perceived benefits and perceived barriers, thereby providing evidence-based support for precision nursing interventions.

**Methods:**

A total of 15 liver cancer patients who underwent TACE at a tertiary A-Grade hospital in Chongqing, China, from June to September 2025 were selected through purposive sampling and participated in semistructured interviews. Themes were identified using Colaizzi’s seven-step method for data analysis.

**Results:**

Five main topics and 13 subtopics were identified: physical barrier perception (postoperative acute symptom burden, gastrointestinal dysfunction, and decreased exercise tolerance), psychological barrier perception (feeling of helplessness, self-perceived burden, and emotional overflow), social support barrier perception (insufficient caregiver accessibility and financial burden), informational-cognitive gap perception (treatment efficacy misconception and low health education accessibility) and rehabilitation benefit perception (physical function improvement, social support benefit, and prior experience benefit).

**Conclusion:**

Liver cancer patients who underwent TACE exhibit multidimensional trade-offs in their perceptions of the benefits and barriers related to health behavior implementation. Healthcare professionals should develop targeted health-behavior intervention strategies based on patients’ perceived characteristics in the early postoperative phase, helping them optimize their physical and psychological well-being and establishing a cognitive foundation for long-term treatment adherence following discharge.

## 1 Introduction

Liver cancer is among the malignant tumors that impose a heavy burden on public health systems. According to a 2024 report in the *Chinese Journal of Epidemiology* (1), liver cancer is among the 6 most common malignant tumors and is the third leading cause of cancer-related death. The International Agency for Research on Cancer, a part of the WHO, reported that approximately 870,000 new liver cancer cases and 760,000 liver cancer-related deaths occurred in 2022 (2). Notably, most patients are diagnosed when the disease is at an intermediate or advanced stage (Stage III–IV), likely because of the progressive nature of liver cancer and the limited efficacy of traditional treatments (3).

Transarterial chemoembolization (TACE) can be used to precisely locate and embolize the blood supply arteries of tumors via catheterization. It can inhibit blood flow and facilitate the slow release of chemotherapeutic agents in the tumor area, thereby promoting continuous tumor necrosis (4–6) and prolonging the survival of liver cancer patients. Thus, TACE is considered the primary treatment for patients with intermediate and advanced liver cancer (4, 7). However, cancer-related pain, TACE-induced pain, postoperative complications, lifestyle changes, and repeated hospitalizations not only exacerbate physical symptoms but also trigger anxiety, depression, and post-traumatic stress responses. These responses decrease compliance with health behaviors and affects long-term prognosis and quality of life (8). Existing research has largely emphasized objective outcome measures such as symptom burden and quality of life among liver cancer patients after TACE (9, 10), as well as descriptive investigations of self-management behaviors, including diet regulation, activity management, and symptom monitoring (11, 12). In contrast, fewer studies have explored—using patients’ own perspectives—the subjective experiences and dynamic decision-making mechanisms underlying patients’ maintenance of or disengagement from postoperative health behaviors. Although nursing intervention studies have provided some practical strategies for patients following TACE, many have not sufficiently incorporated patient-identified perceived needs into the design, leading to limited tailoring. Fundamentally, these issues arise from a methodological neglect of dynamic processes and inadequate probing of patients’ underlying decision-making logic.

The Health Belief Model (HBM) posits that an individual’s perceived susceptibility, perceived severity, perceived benefits, and perceived barriers directly shape health behavior intentions and execution (13). Therefore, eliciting patients’ “benefits versus harms” perceptions from a patient-centered standpoint may offer critical insights for developing precision-based health behavior intervention strategies. Guided by the HBM as a heuristic framework (14), the perceived conflicts and decision dilemmas regarding health behaviors among liver cancer patients after undergoing TACE were thoroughly examined in this descriptive qualitative study. The findings are intended to provide theoretical support for the subsequent development of evidence-based, individualized nursing interventions targeting health behavior.

## 2 Materials and method

### 2.1 Experimental design

A descriptive qualitative research design was employed in this study. Guided by the HBM as a heuristic framework, data were collected through semistructured interviews.

### 2.2 Theory

The HBM was used as a heuristic framework to inform the development of the semistructured interview guide. The model posits that perceived benefits and perceived barriers are core constructs driving or constraining individuals’ decision-making behavior regarding their health. It can effectively explain the cognitive mechanisms underlying patients’ adherence to, or discontinuation of, health behaviors (13). Accordingly, an interview guide was constructed based on the core perspectives of the HBM to comprehensively elicit hepatocellular carcinoma patients’ post-TACE experiences of both positive and negative perceptions related to health behaviors.

### 2.3 Sampling and setting

A purposive maximum-variation sampling strategy was adopted to recruit patients with hepatocellular carcinoma who had undergone TACE at a tertiary Grade A hospital in Chongqing, China, between June and September 2025. To ensure sample representativeness and information sufficiency, participants with diverse characteristics—including age, sex, educational level, caregiver status, disease duration, and the number of TACE treatment sessions—were included. During recruitment, heterogeneity across potential subgroups was intentionally considered to ensure coverage of key participant profiles. The following inclusion criteria were used: ➀ age ≥ 18 years; ➁ clinical or pathological diagnosis meeting the *Guidelines for the Diagnosis and Treatment of Primary Liver Cancer (2024 Edition)*, undergoing TACE treatment for the first time or as a repeat procedure, with postoperative recovery ≥48 h and stable vital signs; ➂ clear consciousness and unimpeded communication; ➃ informed about their own condition; and ➄ voluntary participation and cooperation with the study protocols, with the patient or their family fully informed of the study’s content. The exclusion criteria were as follows: ➀ combined with other malignant tumors or metastatic tumors; ➁ severe cognitive impairment or communication barriers; ➂ end-stage organ failure involving the heart, lungs, liver, kidneys or other organs; and ➃ participation in other clinical trials that were deemed to potentially interfere with health behavior decisions.

### 2.4 Interview topic guide

The research team conducted a systematic search of the literature published between 2000 and 2025 in major Chinese and international databases, including the CNKI, Wanfang Database, the China Medical Literature Database, PubMed, the Cochrane Library, Web of Science, CINAHL, Embase, PsycINFO, and Scopus. The search focused on studies related to health behaviors of hepatocellular carcinoma patients after TACE and patients’s perceived experiences. Drawing on the retrieved evidence, we operationalized postoperative health behaviors as follows: (1) symptom monitoring behaviors, such as monitoring body temperature, abdominal pain and distension, nausea and vomiting, and urine output; (2) postural and activity behaviors, including maintaining lower-limb bed rest following puncture and resuming ambulation in a stepwise manner; (3) dietary management behaviors, including smaller, more frequent meals, a high-protein and low-fat diet, and regulating fluid intake; (4) medication adherence behaviors, such as consistently taking hepatoprotective and antiviral medications; (5) puncture-site care, including keeping the wound clean and dry and monitoring for bleeding and hematoma; and (6) psychological adjustment and information seeking, including proactively reporting physical discomfort, promptly informing healthcare professionals of changes in symptoms, and, using the HBM as a heuristic guiding framework, developing an initial pool of interview elements. A preliminary interview guide was then formed through discussion among research group members and expert consultation.

Two participants who met the inclusion criteria were subsequently selected for pre-interviews. Based on the interview period, information redundancy, and interviewee comprehension, adjustments were made to improve the language. Questions related to “perceived conflict” were added, and the final interview outline was constructed as follows: (1) What precautions did medical staff emphasize to you after your TACE procedure, both in and out of the hospital? (2) What factors hindered you in following these precautions? (3) What perceivable benefits did adherence to these precautions bring you? (4) What personal, family, or medical factors motivated you to consistently adhere to these precautions? (5) On a scale of 0–10, how confident are you in maintaining these health behaviors moving forward, and what are the primary reasons for any reduction in confidence? (6) What additional support would you require from the hospital, community, or family to better facilitate your postoperative health behaviors?

The two participants involved in the pre-interviews were used only to improve and refine the semistructured interview guide. They were not included in the final study sample and did not participate in any subsequent data coding or analysis.

### 2.5 Data collection

The interviewees were contacted in advance to schedule the interviews. On the day of the interview, the research background, purpose, and significance to the interviewees were explained in person, and the interviewees were assured of confidentiality by using codes instead of their real names. Each interview lasted approximately 20–30 min and was conducted in a private meeting room. Given that most participants were older and had lower levels of educational attainment, the Chongqing dialect was chosen to reduce communication barriers and obtain more authentic interview data. With the interviewee’s consent, the entire session was recorded. The interviewer listened attentively without offering suggestions or guidance and focused on the six open-ended questions. The interviewer encouraged the interviewees to fully express their thoughts, employing “paraphrase-clarify-probe” techniques to explore their responses until no new topics arise. Additionally, non-verbal cues, such as facial expressions, body language, and emotional states, were observed and documented during each interview.

Within 24 h after each interview, the audio recordings were transcribed and cross-checked by the research team. The complete raw interview content was then converted into standardized written Mandarin Chinese. Any unclear or ambiguous information was clarified by contacting the participants. Data collection was conducted from June 5, 2025, to September 17, 2025. After interviews 13 and 14 were completed, the coding analysis produced no new themes or subthemes, and the data were considered to have achieved preliminary thematic saturation. To further verify the stability of saturation, a validation interview was conducted with the 15th participant. The additional data also did not generate any new coding categories or research insights. Sampling was then terminated, and the study ultimately included 15 participants.

### 2.6 Data analysis

After all the interviews were transcribed, the data were managed, coded, and analyzed thematically using NVivo 14.0. Data analysis followed Colaizzi’s seven-step method (15): (1) all the interview transcripts were read carefully; (2) significant statements were extracted in relation to the research purpose, and a thematic framework was formulated; (3) statements related to perceptions of health behaviors were progressively extracted and coded; (4) all the coding results were summarized, similar concepts were integrated, and the results were organized into categories; (5) the descriptions of the themes, subthemes, and viewpoints were continuously refined; (6) the concepts across similar viewpoints were identified and elevated to generate higher-level theme meanings; and (7) the interview findings were returned to the participants to confirm whether the results matched their lived experiences, which were revised when necessary to ensure credibility and authenticity.

Overall, the study adopted a bottom-up inductive coding approach, strictly deriving concepts and theme abstractions from the original interview texts to reflect, to the greatest extent possible, patients’ true subjective perceptions and post-procedure behavioral experiences. Coding was conducted independently by two nursing graduate students who had received systematic training in qualitative research. Throughout the coding process, the two coders did not communicate with each other and made independent judgments to minimize subjective bias. After both coders completed the initial coding, all the coded segments and the results of the theme categorization were cross-checked. For any discrepancies in coding interpretations, conceptual boundaries, or theme assignment, all members of the research team revisited the original transcripts and discussed the issues collectively until a consensus conclusion was reached. Throughout the entire analysis process, interview audio recordings, standardized transcripts, multiple rounds of coding files, and records of team discussions and revisions were preserved to create a complete and traceable audit trail. This ensured that the data analysis procedures were standardized and transparent and that the study findings could be reviewed and verified.

### 2.7 Rigor

Several methods were employed in this study to enhance reliability and validity: (1) All the researchers received training in qualitative study methods and had extensive experience in this field. The interviewers were not involved in the clinical diagnosis, treatment, or daily nursing care of the participating patients. The interviewers had no prior or personal/work relationships with the participants, thereby minimizing potential bias; (2) an initial interview outline was drafted based on a literature review, followed by discussions, expert consultations, and pre-interviews to ensure the relevance and accuracy of the interview questions, thereby improving study reliability; (3) strong relationships were established with the interviewees prior to data collection, and the interview durations were extended as needed based on the interview context; and (4) within 24 h of the interview, the recording was reviewed multiple times and transcribed into text data, which were then returned to the interviewees for verification to further enhance the reliability of the data.

### 2.8 Ethics

This study was conducted in accordance with the Helsinki Declaration and received approval from the Hospital Ethics Committee (IRB: number 2022 (326) on December 30, 2022). Researchers received training to ensure that they were competent in conducting the study. Before data collection, all participants were informed about the purpose, procedures, and potential risks of the study. Written informed consent was obtained from each participant prior to the start of the interviews. Audio recordings of the interviews were made following standardized protocols. Participants had the right to withdraw from the study at any time without facing any consequences.

## 3 Results

A total of 15 participants—9 males and 6 females—with ages ranging from 43 to 75 years and an average age of 59 years were included. Among these participants, 13 (86.7%) had an education level of middle school or below. Additionally, 14 (93.3%) had medical insurance. Six participants (40%) had been diagnosed with liver cancer for more than 1 year, and 14 (93.3%) were also diagnosed with hepatitis B or C. Furthermore, 8 participants (53.3%) had experienced two or more sessions of TACE, and 12 (80%) were accompanied by a caregiver. The basic information of the interviewees is summarized in Table 1.

**TABLE 1:** Basic information of the interviewees (*n* = 15).

ID	Age/years	Gender	Education	Medical insurance	Course of liver cancer	Number of TACE	Combined Targeted and Immuno- therapy (Yes/No)	BCLC stage	Child-Pugh class	ECOG	TACE type	Comorbidities	Caregiver
N1	72	M	Primary school	Resident medical insurance	6 months	1	No	B	A	0	DEB-TACE	Hepatitis B virus, Hypertension	Spouse
N2	61	M	Middle school	Employee medical insurance	3 years	5	Yes	B	B	0	cTACE	Hepatitis C virus	Spouse
N3	53	M	Primary school	Resident medical insurance	4 months	1	No	B	A	0	DEB-TACE	Hepatitis C virus	No
N4	72	F	Primary school	Resident medical insurance	2 weeks	1	No	B	A	0	DEB-TACE	Hepatitis B virus, Hypertension	Spouse
N5	75	M	Primary school	Resident medical insurance	1 week	1	No	B	A	0	DEB-TACE	Hepatitis B virus, Hypertension	Grandson
N6	50	M	Primary school	Resident medical insurance	2 years	4	Yes	B	B	1	cTACE	Hepatitis B virus, Hypertension	No
N7	51	F	Middle school	Employee medical insurance	1 month	1	No	B	A	0	DEB-TACE	Hepatitis B virus	Spouse
N8	43	F	Middle school	Employee medical insurance	1 year	2	No	B	A	0	DEB-TACE	Hepatitis B virus	Spouse
N9	56	M	Middle school	Employee medical insurance	2 years	3	Yes	B	A	0	DEB-TACE	Hepatitis B virus	Spouse
N10	67	M	Middle school	Employee medical insurance	8 months	3	Yes	B	A	1	cTACE	Hepatitis B virus, Hypertension	No
N11	52	F	Middle school	Employee medical insurance	2 weeks	1	No	B	A	0	DEB-TACE	Hepatitis C virus	Daughter
N12	46	F	Middle school	Employee medical insurance	3 years	2	No	B	A	0	DEB-TACE	Hepatitis C virus	Nephew
N13	74	F	Illiteracy	Self-funded	2 weeks	1	No	B	A	0	DEB-TACE	Hypertension, diabetes	Daughter
N14	52	M	High school	Employee medical insurance	8 months	3	Yes	B	A	0	DEB-TACE	Hepatitis B virus	Spouse
N15	62	M	High school	Employee medical insurance	7 years	7	Yes	B	B	1	cTACE	Hepatitis B virus	Spouse

Five topics were identified: physical barrier perception, psychological barrier perception, social support barrier perception, informational–cognitive gap perception and rehabilitation benefit perception, as shown in Table 2.

**TABLE 2:** Topics and subtopics.

Topic	Subtopics
Physical barrier perception	Postoperative acute symptom burden
	Gastrointestinal dysfunction
	Decreased exercise tolerance
Psychological barrier perception	Feeling of helplessness
	Self-perceived burden
	Emotional overflow
Social support barrier perception	Insufficient caregiver accessibility
	Financial burden
Informational-cognitive gap perception	Treatment efficacy misconception
	Low health education accessibility
Rehabilitation benefit perception	Physical function improvement
	Social support benefit
	Prior experience benefit

### 3.1 Topic 1: physical barrier perception

#### 3.1.1 Postoperative acute symptom burden

Eight participants (N1, N2, N5, N6, N7, N8, N11, and N15) reported difficulties in implementing health behaviors as instructed by medical staff because of the acute symptom burden following TACE. The most commonly mentioned symptom was post-embolization abdominal pain. This pain is not only extremely uncomfortable but also serves as a severe negative physical and psychological stimulus, ranking among the most intolerable symptoms experienced by patients after TACE. Consequently, it significantly hinders their ability to engage in postoperative health behaviors.

One interviewee described their experience: “*I’m experiencing a burning, wave-like pain in my liver area, and it seems to follow a pattern. It hurts at midnight, then again at 3 a.m., and once more around dawn. As a result, my sleep has been severely affected, and I only get about 1–2 h of rest each night.*” (N1)

Another shared a more distressing account: “*The pain in my abdomen is excruciating. I need painkiller injections, but they only last for 8 h. Apart from those few hours of relief, it’s pure misery, and I can do nothing. Sometimes… sometimes I considered suicide. (At this point, the interviewee broke down in tears.)*” (N11)

#### 3.1.2 Gastrointestinal dysfunction

Eight participants (N1, N3, N4, N5, N6, N9, N12, and N15) reported experiencing gastrointestinal dysfunction after TACE treatment, which primarily manifested as an aversion to fatty foods, abdominal distension, nausea, and constipation. These symptoms hinder their ability to eat normally and replenish nutrients.

One participant noted, “*In the first 2 days following the surgery, I developed an intolerance to greasy food, which triggered nausea and a complete barrier to my appetite. As a result, I experienced persistent stomach discomfort throughout that period.*” (N4)

Another shared, “*Another issue is constipation. I can’t pass stool without enemas. This is really distressing. I’m so afraid to eat because eating makes me feel bloated. But if I don’t eat, it becomes even harder to have a bowel movement. It’s a vicious cycle!*” (N9)

A third participant explained, “*I eat very little. First, I have no appetite; I actually have to force myself to eat to survive. Secondly, my abdomen feels extremely bloated, and that sensation worsens if I eat something. As a result, I don’t dare to eat more*.” (N12)

#### 3.1.3 Decreased exercise tolerance

Seven participants (N1, N2, N5, N6, N12, N14, and N15) reported symptoms such as fatigue and decreased exercise tolerance following TACE treatment compared with their preoperative state.

“*Apart from dizziness, I feel somewhat weak, especially when getting out of bed. My grandson has to support me, otherwise, my legs feel weak and I can only walk a few steps.*” (N5)

“*I seldom get out of bed, mainly because I feel exhausted and extremely tired. My legs feel like soft clay that can barely move*.” (N12)

### 3.2 Topic 2: Psychological barrier perception

#### 3.2.1 Feeling of helplessness

The repeated cycle of “readmission—waiting for follow-up examinations—repeated TACE” kept patients in a prolonged state of being constantly reminded of their illness. Postoperative complications (e.g., acute pancreatitis and post-embolization syndrome) further weakened their sense of control, leading to negative psychological experiences such as anxiety and depression. Eight participants (N2, N3, N7, N10, N11, N13, and N14) expressed feelings of helplessness regarding their disease and uncertainty about recovery.

“*Sometimes I wonder why I’m the unlucky one. I was diagnosed with this disease, underwent surgery, and then developed pancreatitis as a complication. The doctors assured me that this complication is rare, yet here I am. Now, I rely on a painkiller pump every day, connected to machines, unable to get out of bed. I spend my days lying here, and at times, I feel like it would be better to die.*” (N2)

“*My sleep is poor now. It’s not physical discomfort that prevents me from resting. As soon as I lie down, my mind starts racing: Why did I get this illness? Will I be able to recover? I find myself overthinking everything, and this mental turmoil keeps me from sleeping.*” (N10)

#### 3.2.2 Self-perceived burden

Long-term and repeated TACE treatment made patients highly sensitive to “care dependence.” In general, patients developed self-blaming cognitions such as “being a burden to the family,” and these perceptions further intensified as the caregiving burden increased. This viewpoint was mentioned by nine participants (N1, N2, N3, N7, N10, N13, N14, and N15).

“*Sometimes I feel so guilty toward my wife. She has been so busy taking care of me since I got sick. We took a bus to the hospital, and she had to carry all the bags while enduring terrible carsickness. She does most things for me, even though she isn’t in good health herself. Her shoulders and back often hurt. Well, what can we do? (Sighs).*” (N1)

“*Sometimes I feel deeply depressed, wondering why I got this disease in my early fifties. I also feel like a burden to my family. My eldest son got married 2 years ago, and my younger daughter is only eleven. If I weren’t sick, I could take her out during the summer vacation. But now, her mother has to care for me in the hospital, so my daughter has to come here every day to keep me company.*” (N2)

Three participants (N2, N7, and N11) interviewees, driven by intense self-blame and guilt, developed negative thoughts such as “they would be better off without me,” which severely affected their treatment compliance.

“*Right now, my daughter is taking care of me. She is a college student, but her semester hasn’t started yet. Once the semester begins in a few days, I’ll have to take care of myself. We have no choice in this matter. My husband will work to financially support the family. Sometimes, I feel like a burden to them, and I consider just stopping treatment. To be honest, that thought comes to me very frequently (She said with a bitter smile)*.” (N11)

#### 3.2.3 Emotional overflow

Postoperative multidimensional stressor stimulation after TACE markedly reduced patients’ emotional regulation thresholds, resulting in an “irritability–hostility” extroverted emotional overflow. Five interviewees (N3, N5, N9, N11, and N14) reported that they experienced intense emotional fluctuations postoperatively, manifesting as verbal confrontations with family members or medical staff.

“*I used to have a really good temper and never lost my temper with anyone. But since I got the disease, I have heard repeated episodes of temper during my constant visits to the hospital for embolization therapy. I am unable to concentrate and follow the doctors’ instructions. I often think they are just exploit me for money, so I end up treating them very badly.*” (N6)

“*Ever since I was admitted for surgery, my daughter has been telling me, “Mom, you are sometimes harsh recently, you lose your temper so easily.” But I can’t help it. I have no control over it. When the misery and frustration build up, I have such a short fuse and just explode*.” (N11)

### 3.3 Topic 3: social support barrier

#### 3.3.1 Insufficient caregiver accessibility

Three participants (N3, N6, and N10) reported that their primary caregivers were unable to remain at the bedside continuously because they had to balance multiple responsibilities simultaneously. When caregiving support was reduced, patients needed to complete portions of their daily care by themselves at an earlier stage, including getting out of bed, eating, and observing and reporting their own symptoms.

“*I’m taking care of myself. I was out of bed the day after the surgery, and as soon as I could manage, I told my wife to go back home. We don’t live in this city, and our house is far away. If she stays, housework would be unattended.*” (N3)

“*I have no caregivers. My wife returns home to take care of our grandson. I still find it difficult to navigate, but what choice do I have? At noon, I make my way downstairs to buy lunch, getting enough to save for dinner.*” (N10)

#### 3.3.2 Financial burden

Eleven participants (N1, N2, N3, N5, N6, N7, N8, N12, N13, N14, and N15) noted that while TACE was more affordable than other treatment options were, the costs associated with repeated procedures still created a significant financial burden.

“*What worries me most now is the treatment cost. I haven’t been able to work since I got sick, so my husband is bearing all the household expenses. The kids also support us, but my daughter just started working not long ago, and her pay is very low. This disease puts an enormous strain on the family (Sighs).*” (N7)

Two participants (N3 and N6) mentioned that they had to return to work immediately after discharge, which made it challenging to adhere to medical advice regarding health behaviors.

“*Recovery requires money. The nurses recommend a light, nutritious diet, but we’re construction workers, and we eat the standard site meals. With our second child as a college student, how could I possibly afford to eat separately? The doctors say I need long-term treatment, medication, and follow-ups, and I should avoid heavy labor. If that’s the case, how do we get the money for our daily lives?… It’s so worrying!*” (N3)

“*I’ll go back to work immediately after discharge. I’m not married and have no children, so I have to handle everything on my own. I’ll have to push myself to act like a normal person. Otherwise, how would I afford my living expenses and treatment?*” (N6)

### 3.4 Topic 4: Informational–cognitive gap

#### 3.4.1 Treatment efficacy misconception

Five participants (N4, N5, N6, N9, and N13) exhibited an excessive focus on changes in clinical symptoms, which led them to overlook the significance of established treatment regimens. This tendency often resulted in self-discontinuation of medication and questioning of the prescribed therapeutic regimen.

“*Do I still have to continue taking medication after discharge? I’ve been on that antiviral medicine for over 10 years, and it is the last thing I want right now. Previously, they said the medicine can prevent my hepatitis B from worsening and turning into liver cancer. Now that I’ve had the surgery, can I stop the medication or at least reduce the dose after discharge?*” (N4)

“*I don’t have any issues other than swollen legs. Sometimes it gets so bad that I can’t even get my shoes on. How can I get out of bed to exercise? I’ve told the doctor, and after following his treatment for a few days, it gets better, but then the swelling returns. It doesn’t seem very useful. I might just take some diuretics*.” (N6)

“*After this surgery, I didn’t have the same abdominal pain as before, which makes me worried about the efficacy. Didn’t they say that after embolizing the blood vessels, the bad cells inside me would die, causing stomach pain? Why didn’t that happen this time? Was the efficacy not good enough? Will the cancer come back soon?*” (N9)

#### 3.4.2 Low health education accessibility

Seven participants (N1, N3, N4, N5, N6, N9, and N13) with low levels of education struggled to accept the health education provided by nurses partly because of widespread misinformation online.

One interviewee explained, “*I haven’t been doing a great job following the instructions from the nurses. My education is low, and sometimes they use professional terms that I don’t understand. Additionally, the doctors and nurses on the ward are so busy that I feel bad about asking them to repeat things for me. As a result, I sometimes turn to WeChat public accounts for help.*” (N3)

Another interviewee asked, “*Aside from interventional therapy, immunotherapy, and targeted therapy, does your hospital offer any more advanced treatments? I heard about a treatment that can cure any cancer, but it is not accessible to ordinary people like us. Is that true?*” (N6)

### 3.5 Topic 5: Rehabilitation benefit perception

#### 3.5.1 Physical function improvement

Nine participants (N1, N3, N4, N7, N8, N10, N12, N14, and N15) indicated that actively practicing healthy behaviors after surgery significantly aided in their recovery.

One participant shared, “*Now that I’m recovering, I go downstairs for a walk with my wife every evening after dinner or during cool mornings. Staying in a small room all day is unbearable. Since I’ve been walking regularly, I feel that my sleep has improved greatly. Additionally, the constant bloating and discomfort I experienced right after the surgery have eased, and my bowel movements are now much more regular*” (N1)

Another participant noted, “*I feel great now, with no discomfort. Right after the surgery, my abdomen was very bloated, and I had no appetite. However, the doctor said I could get out of bed the next day. After I moved around a bit and passed gas, I felt much better. Therefore, I truly believe that the advice you gave me about continuing to exercise after discharge is essential!*” (N3)

#### 3.5.2 Social support benefit

Ten participants (N1, N2, N4, N6, N7, N8, N10, N11, N12, and N14) mentioned that the emotional support and practical caregiving provided by family members helped them feel more resilient when dealing with postoperative discomfort and made them more willing to comply with health behaviors recommended by healthcare professionals.

“*Every time I come to the hospital, to be honest, I feel so depressed, especially when I’m dealing with disease symptoms. But my wife encourages me to withstand the treatments. Without her, I wouldn’t have made it this far. Because of her, no matter how tired or unwell I feel, I’m determined to follow your instructions carefully (Cry).*” (N2)

“*Both daughters are very filial. My eldest daughter is quite busy, but she often visits me. Currently, it’s my younger daughter who takes care of me. She stays with me until my IV drip is finished and then rushes off to buy me a meal. I must eat well, of course, to worry them less*.” (N13)

#### 3.5.3 Prior experience benefit

Three participants (N8, N12, and N15) who had previously undergone TACE mentioned that their previous experiences helped them understand more clearly what they needed to pay attention to after surgery. As a result, they were more willing to adhere to relevant health behaviors during this postoperative period.

“*This surgery went a bit better for me than the last one. Since it is the second one for me, I was more aware of all the precautions and knew what to do*.” (N8)

“*Although I still don’t have much appetite after this surgery, my experience last time told me that I can manage it by eating smaller meals more frequently. I should start with an amount that won’t trigger severe abdominal distension or pain, and then gradually increase it. This approach also helps me slowly begin getting out of bed*.” (N12)

## 4 Discussion

### 4.1 Main findings

This study revealed a dynamic trade-off between “barrier” and “benefit” perceptions in the adoption of health behaviors among liver cancer patients following TACE. The perception of acute symptom burden, caregiver accessibility, financial strain, and informational–cognitive gaps form the “barrier perception” in the HBM framework. These perceived barriers are further intensified by negative psychological responses—such as anxiety, helplessness, and self-perceived burdensomeness—thereby increasing the difficulty of engaging in health behaviors. In contrast, positive experiences—such as improvements in physical function, social support, and prior experience benefit—strengthen the “perceived benefits,” promoting internalization and sustained adherence to health behaviors.

Previous studies have shown that postoperative symptoms are the primary barrier to compliance with health behaviors (11, 16–18). In this study, symptom burden was identified as the primary factor influencing barrier perception. The research also highlights the interconnected mechanisms among symptoms, psychological factors, and social support, enhancing our understanding of the relationship between “barrier perception–psychological burden–behavioral avoidance.” Additionally, the study revealed significant shortcomings at the information level, including misconceptions about treatment efficacy and limited access to health education. This underscores the necessity of reshaping patients’ efficacy evaluation frameworks through nursing education and addressing knowledge gaps with clear and accessible methods.

### 4.2 Inspiration for nursing practice

#### 4.2.1 Enhancing symptom management to improve the ability of liver cancer patients to implement health behaviors after TACE

Post-TACE embolization syndrome, which includes symptoms such as pain, nausea, vomiting, and fever, is the primary source of “barrier perception” in patients. This syndrome is a key trigger for the imbalance in the “behavior benefits/barrier trade-off” within the HBM. Additionally, in accordance with Maslow’s hierarchy of needs, physiological needs form the foundation of all human needs (19). Addressing symptoms in liver cancer patients after TACE and implementing targeted intervention strategies are essential responsibilities for medical staff. Existing guidelines and expert consensus indicate that the incidence of post-embolization syndrome is approximately 40%–80% (20), and it is usually self-limiting; symptoms can typically be effectively alleviated within 1–3 days through symptomatic supportive care. Nursing staff should closely monitor patients’ vital signs and symptom changes after surgery and promptly explain the causes of symptoms to reduce patients’ uncertainty and anxiety. In addition, nurses should implement evidence-based symptom management, including dynamically assessing pain and providing stepwise analgesia (21, 22), guiding patients to consume small, frequent meals and adopt bland, easily digestible diets to reduce gastrointestinal irritation (23), and monitoring temperature changes while offering appropriate antipyretic and fluid support (24). With effective symptom control, patients’ perceived barriers to health behaviors can be reduced, facilitating a shift from “passive endurance” to “active participation.”

#### 4.2.2 Alleviating caregiver role overload and financial burden and rebuilding a social support network

The social support barrier identified in this study appeared as “insufficient caregiver accessibility” and “financial burden,” which intertwined and compounded one another. Patients were required to manage self-care within 48 h post-surgery and to prematurely end rehabilitation training, not because of emotional detachment but because of role overload and repeated TACE treatments. Although repeated TACE is relatively low-cost, it still leads to cumulative out-of-pocket expenses following income disruption, forcing families to make difficult choices between “caring for the patient” and “sustaining the household.” Therefore, clinical teams should focus on the structural causes underlying social support impairment rather than simply attributing it to a lack of family affection. In nursing practice, caregiver burden assessment and referral to social resources should be integrated into routine supportive care workflows. By early identification of high-risk families (25) and coordination of multidisciplinary supportive services, nurses can reduce patients’ dilemmas in choosing to interrupt rehabilitation because of support deficits.

#### 4.2.3 Effectively managing emotions and rebuilding psychological stress mechanisms in liver cancer patients after TACE

After TACE, patients face the combined physiological stress of their illness and the effects of interventional surgery, along with psychological pressure from feeling like a burden to their families. This is compounded by the uncertainty of a prolonged recovery process (18). As a result of these multiple stresses, psychological burden has emerged as a key component of “health behavior barrier perception.” It amplifies patients’ fear of complications and induces avoidance, irritability and other stress responses, accompanied by anxiety, depression and insomnia (26), directly reducing post-surgery health behavior compliance. Therefore, in addition to prioritizing patients’ physical health, nurses should place greater emphasis on their mental health. They should offer professional counseling and guidance in the family environment, encouraging family members to provide adequate emotional support. Emphasizing the crucial role of family members, particularly caregivers, in the patient’s postoperative recovery process is essential. This approach helps patients gain a clearer understanding of their physical condition while alleviating their psychological burden. Nurses can utilize self-management and coping strategies, along with cognitive behavioral therapy, mindfulness, and stress management techniques, to help patients achieve psychological adjustment (26, 27). These approaches effectively reduce negative emotions and psychological pressure while simultaneously increasing patients’ confidence in adopting health behaviors.

#### 4.2.4 Building evidence-based accurate health education to address treatment efficacy misconceptions

Patients with chronic diseases frequently use the internet as their primary information source because of uncertainty about therapeutic regimens, fear of disease progression, and the need for shared decision-making (28). Patients with lower levels of education are particularly vulnerable to the influence of misinformation. This vulnerability can lead to inaccurate estimates and uncertainty regarding recurrence and complication risks, which in turn may cause patients to question their treatment plans and exhibit decreased compliance. According to the HBM, perceived susceptibility directly affects the motivation to adopt protective behaviors. Therefore, before implementing health education, nurses should systematically assess patients’ health literacy and information needs, focusing on their specific concerns and knowledge gaps. Personalized education should be provided to help patients understand their condition while guiding them to establish a comprehensive and objective framework for evaluating treatment efficacy rather than relying solely on clinical symptom relief as a criterion. By offering continuous and accurate health education, nurses can reduce patients’ uncertainty about their treatment regimens and alleviate fears related to disease progression. This approach ultimately enhances treatment compliance and encourages the internalization and maintenance of healthy behaviors.

### 4.3 Limitations

This study has several limitations. First, it is a single-center, small-sample study. The overall educational level of the interviewed patients was relatively low, and the participants included mainly individuals with hepatitis-related hepatocellular carcinoma. These characteristics make the study population somewhat specific, which restricts the generalizability and the scope of transfer of the findings. In addition, subgroup comparisons based on disease course, number of treatment sessions, educational level, or disease severity, were not conducted in this study, thereby limiting the external validity of the conclusions. Second, the timing of the interviews was uniformly set to the inpatient recovery phase which was 48 h after TACE or later. At this stage, patients are still in the postoperative stress adaptation period. Therefore, the perceived experiences and behavioral intentions observed in this study may strongly reflect patients’ early postoperative psychological states and short-term recovery cognition, making it difficult to fully capture health behavior adherence during long-term home-based recovery after discharge. This creates a degree of temporal limitation. Third, the study included only the patients’ perspective. It did not triangulate the findings with caregivers’ caregiving experiences or with assessments from clinical physicians and nurses. As a result, the perceptions covered in this study are relatively one-dimensional. Moreover, all the data were obtained through patients’ self-reports, which inevitably introduces self-report bias. Based on these limitations, future research can be strengthened by conducting multicenter, large-sample, longitudinal follow-up studies and increasing the number of participants recruited across regions, educational levels, etiologies, and disease characteristics. Such studies should dynamically track the long-term trajectories of patients’ postoperative health cognition and behavior. Moreover, integrating data from multiple perspectives—patients, caregivers, and healthcare professionals—can increase the dimensionality of the evidence. Building on this finding, researchers may develop a standardized instrument, such as a “Decisional Balance Scale for Health Behaviors after TACE in Hepatocellular Carcinoma Patients,” providing a useful tool for evaluating the effectiveness of precision interventions.

## 5 Conclusion

Guided by the HBM as a heuristic framework, qualitative methods were used in this study to explore the perceptions of hepatocellular carcinoma patients when health behaviors were implemented after TACE. Five core topics were identified, namely, physical barrier perception, psychological barrier perception, social support barrier perception, informational–cognitive gap perception and rehabilitation benefit perception. These themes are interrelated and mutually influencing, jointly shaping patients’ early treatment adherence and quality of life after TACE. Nursing staff should conduct targeted assessments based on patients’ early postoperative perceptions, and mobilize resources from the individual, family, healthcare system, and society to construct early intervention plans. This can help patients establish an initial sense of recovery-related behavioral awareness, optimize their postoperative physical and psychological rehabilitation status, and lay an important cognitive foundation for maintaining health behaviors and improving long-term treatment adherence after discharge.

## Data Availability

The original contributions presented in this study are included in this article/supplementary material, further inquiries can be directed to the corresponding author.
